# CPAN - A novel transdiagnostic dimensional approach to the assessment of psychotic disorders

**DOI:** 10.1192/j.eurpsy.2024.1532

**Published:** 2024-08-27

**Authors:** L. Hermán, J. M. Réthelyi, J. Tolna, E. Komoróczy, I. B. Császár, M. Baradits

**Affiliations:** Department of Psychiatry and Psychotherapy, Semmelweis University, Budapest, Hungary

## Abstract

**Introduction:**

Classification of mental disorders evolved greatly over time, as DSM and ICD dominated both research and everyday practice in the past decades. DSM-5 was planned to represent biological features of psychiatric disorders and include results of genetic and imaging studies in the criteria. Unfortunately, this goal couldn’t be fulfilled, since, although there were promising results, evidence wasn’t strong enough to fully support the biological background of the currently used diagnostic categories. One possible explanation for this discrepancy is that biological disturbances don’t represent the somewhat artificial categorisation of these disorders. Many of the leading symptoms in psychotic disorders are nowadays considered as lying on a spectrum, such as autism, affective and psychotic spectrum disorders. Despite that, DSM-5 still describes schizophrenia, schizoaffective disorder and bipolar disorder as separate entities, however there can be major overlaps in the leading symptoms, moreover symptoms are not necessarily stable over time and can show fluctuations. It should be mentioned though that subgroups of schizophrenia in DSM-5 had been abolished and catatonia is considered as a trans-diagnostic specifier, moreover in ICD-11 certain symptoms can be added as symptoms specifiers to an existing diagnosis of primary psychotic disorder.

**Objectives:**

Our aim was to establish a new trans-diagnostic, dimensional scale to assess the most important symptoms amongst patients with psychotic disorders. This scale is meant to represent the long-term clinical presentation and not a cross-sectional picture of a current state. We believe that long-term trajectories of these symptoms may be more connected to underlying biological features, such as genetic load (i.e. polygenic risk scores) and imaging results than the currently used diagnostic criteria. We think it is very important to create a tool, which is straightforward and short enough, so can be realistically used in everyday clinical work. This could provide important real-life data, which give us information about our patients from a different angle than the currently used diagnostic systems.

**Methods:**

We have created the CPAN scale based on the current symptom specifiers of ICD-11 and the Clinician-Rated Dimensions of Psychosis Symptom Severity, which is an “emerging measure” for DSM-5 and also took into consideration our own clinical experience.

**Results:**

The new tool measures 4 symptoms (catatonia, psychosis, affective symptoms and negative symptoms) on a scale of 5 (0-4). We have also put in specifiers to be able to characterize patients more precisely, and output measures (suicidal risk, functionality) to open the way for further analysis.

**Conclusions:**

We tried to establish a novel symptom scale to help assessing patients with psychotic symptoms in everyday clinical work. Our plan is to test the validity of CPAN in the near future.

**Disclosure of Interest:**

None Declared

